# 5-Chloro-3-ethyl­sulfinyl-2-(4-iodo­phen­yl)-7-methyl-1-benzofuran

**DOI:** 10.1107/S160053681003134X

**Published:** 2010-08-11

**Authors:** Hong Dae Choi, Pil Ja Seo, Byeng Wha Son, Uk Lee

**Affiliations:** a Department of Chemistry, Dongeui University, San 24 Kaya-dong Busanjin-gu, Busan 614-714, Republic of Korea; b Department of Chemistry, Pukyong National University, 599-1 Daeyeon 3-dong Nam-gu, Busan 608-737, Republic of Korea

## Abstract

In the title compound, C_17_H_14_ClIO_2_S, the 4-iodo­phenyl ring makes a dihedral angle of 1.61 (9)° with the benzofuran ring system. In the crystal, mol­ecules are linked through a weak inter­molecular C—H⋯O hydrogen bond and an I⋯O contact [3.416 (2) Å]. The ethyl group is disordered over two orientations with site-occupancy factors of 0.402 (7) and 0.598 (7).

## Related literature

For the pharmacological activity of benzofuran compounds, see: Aslam *et al.* (2006 ▶); Galal *et al.* (2009 ▶); Khan *et al.* (2005 ▶). For natural products with benzofuran rings, see: Akgul & Anil (2003 ▶); Soekamto *et al.* (2003 ▶). For the structures of related 3-ethyl­sulfinyl-2-(4-iodo­phen­yl)-1-benzofuran derivatives, see: Choi *et al.* (2010*a* ▶,*b* ▶). For a review of halogen bonding, see: Politzer *et al.* (2007 ▶).
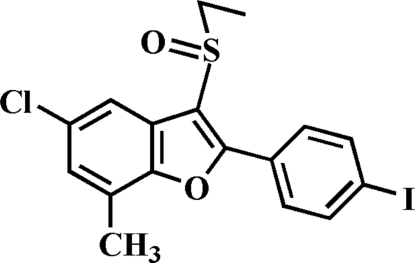


## Experimental

### 

#### Crystal data

C_17_H_14_ClIO_2_S
*M* *_r_* = 444.69
Triclinic, 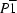

*a* = 7.8013 (2) Å
*b* = 10.4240 (3) Å
*c* = 11.6003 (3) Å
α = 115.962 (1)°
β = 98.040 (1)°
γ = 96.661 (1)°
*V* = 823.00 (4) Å^3^
*Z* = 2
Mo *K*α radiation
μ = 2.24 mm^−1^
*T* = 173 K
0.48 × 0.34 × 0.16 mm

#### Data collection

Bruker SMART APEXII CCD diffractometer
Absorption correction: multi-scan (*SADABS*; Bruker, 2009 ▶) *T*_min_ = 0.473, *T*_max_ = 0.746
14721 measured reflections
3773 independent reflections
3408 reflections with *I* > 2σ(*I*)
*R*_int_ = 0.024

#### Refinement

*R*[*F* ^2^ > 2σ(*F* ^2^)] = 0.024
*wR*(*F* ^2^) = 0.065
*S* = 1.06
3773 reflections
221 parameters
28 restraints
H-atom parameters constrained
Δρ_max_ = 0.28 e Å^−3^
Δρ_min_ = −0.60 e Å^−3^

### 

Data collection: *APEX2* (Bruker, 2009 ▶); cell refinement: *SAINT* (Bruker, 2009 ▶); data reduction: *SAINT*; program(s) used to solve structure: *SHELXS97* (Sheldrick, 2008 ▶); program(s) used to refine structure: *SHELXL97* (Sheldrick, 2008 ▶); molecular graphics: *ORTEP-3* (Farrugia, 1997 ▶) and *DIAMOND* (Brandenburg, 1998 ▶); software used to prepare material for publication: *SHELXL97*.

## Supplementary Material

Crystal structure: contains datablocks global, I. DOI: 10.1107/S160053681003134X/is2588sup1.cif

Structure factors: contains datablocks I. DOI: 10.1107/S160053681003134X/is2588Isup2.hkl

Additional supplementary materials:  crystallographic information; 3D view; checkCIF report

## Figures and Tables

**Table 1:** Hydrogen-bond geometry (Å, °)

*D*—H⋯*A*	*D*—H	H⋯*A*	*D*⋯*A*	*D*—H⋯*A*
C16*A*—H16*B*⋯O2^i^	0.97	2.40	3.297 (6)	154

Symmetry code: (i) 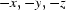.

**Fig. 1.**
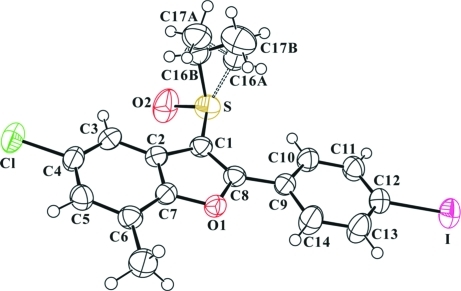
The molecular structure of the title compound with the atom numbering scheme. Displacement ellipsoids are drawn at the 50% probability level. H atoms are presented as a small spheres of arbitrary radius. The ethyl group is disordered over two positions with site-occupancy factors, from refinement of 0.402 (7) and 0.598 (7).

**Fig. 2.**
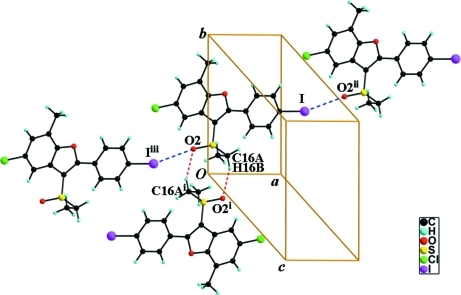
C—H···O and I···O interactions (dotted lines) in the crystal structure of the title compound. The disordered component of the ethyl group, part B, has been omitted for clarity as have H atoms not involved in intermolecular contacts. [Symmetry codes: (i) - x, - y, - z; (ii) x + 1, y + 1, z + 1; (iii) x - 1 , y - 1, z - 1.]

## supplementary crystallographic information

### Comment

Many compounds containing a benzofuran ring have received considerable attention in view of their pharmacological properties such as antifungal, antitumor and antiviral, antimicrobial activities (Aslam *et al.*, 2006, Galal *et al.*, 2009, Khan *et al.*, 2005). These compounds widely occur in nature (Akgul & Anil, 2003; Soekamto *et al.*, 2003). As a part of our continuing studies of the substituent effect on the solid state structures of 3-ethylsulfinyl-2-(4-iodophenyl)-1-benzofuran analogues (Choi *et al.*, 2010*a*, *b*), we report the crystal structure of the title compound (Fig. 1).

The benzofuran unit is essentially planar, with a mean deviation of 0.007 (2) Å from the least-squares plane defined by the nine constituent atoms. The ethyl group is disordered over two positions with site–occupancy factors of 0.402 (7) (for atom labelled A) and 0.598 (7) (for atom labelled B) in Fig. 1. The dihedral angle formed by the benzofuran plane and the 4–iodophenyl ring is 1.61 (9)°. The molecular packing (Fig. 2) is stabilized by a weak intermolecular C—H···O hydrogen bond between the methylene H atom of the ethyl group and the oxygen of the S═O unit, with a C16A—H16B···O2^i^ (Table 1). The crystal packing (Fig. 2) is further stabilized by an I···O halogen-bonding between the iodine and the oxygen of the S═O unit [I···O2^ii^ = 3.416 (2) Å; C12—I···O2^ii^ = 161.46 (7)°] (Politzer *et al.*, 2007).

### Experimental

77% 3-chloroperoxybenzoic acid (157 mg, 0.7 mmol) was added in small portions to a stirred solution of 5-chloro-3-ethylsulfanyl-2-(4-iodophenyl)-7-methyl-1-benzofuran (300 mg, 0.7 mmol) in dichloromethane (40 mL) at 273 K. After being stirred at room temperature for 5h, the mixture was washed with saturated sodium bicarbonate solution and the organic layer was separated, dried over magnesium sulfate, filtered and concentrated at reduced pressure. The residue was purified by column chromatography (hexane–ethyl acetate, 1:1 v/v) to afford the title compound as a colorless solid [yield 76%, m.p. 457–458 K; R/_f_ = 0.64 (hexane–ethyl acetate, 1:1 v/v)]. Single crystals suitable for X-ray diffraction were prepared by slow evaporation of a solution of the title compound in acetone at room temperature.

### Refinement

All H atoms were positioned geometrically and refined using a riding model, with C—H = 0.93 Å for aryl, 0.97 Å for methylene, and 0.96 Å for methyl H atoms, and with *U*_iso_(H) =1.2*U*_eq_(C) for aryl and methylene H atoms, and 1.5*U*_eq_(C) for methyl H atoms. The ethyl  group was found to be disordered over two positions and modelled with site-occupancy factors, from refinement of  0.402 (7) (part A) and  0.598 (7) (part B), respectively. The displacement ellipsoids of part B were restrained using command ISOR (0.01), sets of C atoms were restrained using command DELU and the distances of C—C were restrained to 1.480 (2) Å using command DFIX. The distances of C—S and C—C were restrained to 0.001Å using command SADI.

### Crystal data

**Table:**

C_17_H_14_ClIO_2_S	*Z* = 2
*M**_r_* = 444.69	*F*(000) = 436
Triclinic, *P*1	*D*_x_ = 1.794 Mg m^−^^3^
Hall symbol: -P 1	Mo *K*α radiation, λ = 0.71073 Å
*a* = 7.8013 (2) Å	Cell parameters from 9231 reflections
*b* = 10.4240 (3) Å	θ = 2.2–27.5°
*c* = 11.6003 (3) Å	µ = 2.24 mm^−^^1^
α = 115.962 (1)°	*T* = 173 K
β = 98.040 (1)°	Block, colourless
γ = 96.661 (1)°	0.48 × 0.34 × 0.16 mm
*V* = 823.00 (4) Å^3^	

### Data collection

**Table:**

Bruker SMART APEXII CCD diffractometer	3773 independent reflections
Radiation source: rotating anode	3408 reflections with *I* > 2σ(*I*)
graphite multilayer	*R*_int_ = 0.024
Detector resolution: 10.0 pixels mm^-1^	θ_max_ = 27.5°, θ_min_ = 2.0°
φ and ω scans	*h* = −9→10
Absorption correction: multi-scan (*SADABS*; Bruker, 2009)	*k* = −13→13
*T*_min_ = 0.473, *T*_max_ = 0.746	*l* = −15→15
14721 measured reflections	

### Refinement

**Table:**

Refinement on *F*^2^	Primary atom site location: structure-invariant direct methods
Least-squares matrix: full	Secondary atom site location: difference Fourier map
*R*[*F*^2^ > 2σ(*F*^2^)] = 0.024	Hydrogen site location: difference Fourier map
*wR*(*F*^2^) = 0.065	H-atom parameters constrained
*S* = 1.06	*w* = 1/[σ^2^(*F*_o_^2^) + (0.034*P*)^2^ + 0.2748*P*] where *P* = (*F*_o_^2^ + 2*F*_c_^2^)/3
3773 reflections	(Δ/σ)_max_ = 0.001
221 parameters	Δρ_max_ = 0.28 e Å^−^^3^
28 restraints	Δρ_min_ = −0.60 e Å^−^^3^

### Special details

**Table:**

Geometry. All esds (except the esd in the dihedral angle between two l.s. planes) are estimated using the full covariance matrix. The cell esds are taken into account individually in the estimation of esds in distances, angles and torsion angles; correlations between esds in cell parameters are only used when they are defined by crystal symmetry. An approximate (isotropic) treatment of cell esds is used for estimating esds involving l.s. planes.
Refinement. Refinement of F^2^ against ALL reflections. The weighted R-factor wR and goodness of fit S are based on F^2^, conventional R-factors R are based on F, with F set to zero for negative F^2^. The threshold expression of F^2^ > 2sigma(F^2^) is used only for calculating R-factors(gt) etc. and is not relevant to the choice of reflections for refinement. R-factors based on F^2^ are statistically about twice as large as those based on F, and R- factors based on ALL data will be even larger.

### Fractional atomic coordinates and isotropic or equivalent isotropic displacement parameters (Å^2^)

**Table:**

	*x*	*y*	*z*	*U*_iso_*/*U*_eq_	Occ. (<1)
I	0.64468 (2)	0.843070 (18)	0.633828 (13)	0.05745 (7)	
Cl	−0.13606 (8)	0.12905 (7)	−0.54903 (5)	0.06029 (16)	
S	0.05956 (8)	0.20738 (7)	0.00343 (6)	0.05340 (14)	
O1	0.26320 (19)	0.55334 (15)	−0.03279 (13)	0.0397 (3)	
O2	−0.1151 (3)	0.1273 (2)	−0.0800 (2)	0.0830 (7)	
C3	−0.0084 (3)	0.2242 (2)	−0.2921 (2)	0.0432 (4)	
H3	−0.0663	0.1352	−0.3031	0.052*	
C1	0.1344 (3)	0.3441 (2)	−0.04020 (19)	0.0398 (4)	
C2	0.0905 (3)	0.3316 (2)	−0.17052 (19)	0.0383 (4)	
C4	−0.0153 (3)	0.2577 (2)	−0.3946 (2)	0.0440 (4)	
C5	0.0668 (3)	0.3905 (2)	−0.3815 (2)	0.0450 (5)	
H5	0.0567	0.4065	−0.4547	0.054*	
C6	0.1637 (3)	0.4998 (2)	−0.2612 (2)	0.0413 (4)	
C7	0.1723 (3)	0.4632 (2)	−0.15913 (18)	0.0377 (4)	
C8	0.2378 (3)	0.4790 (2)	0.03875 (18)	0.0384 (4)	
C9	0.3273 (3)	0.5601 (2)	0.17695 (19)	0.0396 (4)	
C10	0.3167 (3)	0.5045 (3)	0.2654 (2)	0.0498 (5)	
H10	0.2503	0.4120	0.2370	0.060*	
C11	0.4045 (3)	0.5857 (3)	0.3958 (2)	0.0512 (5)	
H11	0.3943	0.5481	0.4545	0.061*	
C12	0.5061 (3)	0.7208 (2)	0.4386 (2)	0.0446 (5)	
C13	0.5151 (4)	0.7794 (3)	0.3532 (2)	0.0611 (7)	
H13	0.5815	0.8721	0.3825	0.073*	
C14	0.4253 (4)	0.7001 (3)	0.2240 (2)	0.0590 (6)	
H14	0.4303	0.7410	0.1673	0.071*	
C15	0.2513 (4)	0.6454 (3)	−0.2418 (3)	0.0580 (6)	
H15A	0.3745	0.6468	−0.2421	0.087*	
H15B	0.2385	0.7197	−0.1593	0.087*	
H15C	0.1971	0.6631	−0.3116	0.087*	
C16A	0.2618 (6)	0.1332 (6)	0.0089 (5)	0.0561 (19)	0.402 (7)
H16A	0.3649	0.2108	0.0570	0.067*	0.402 (7)
H16B	0.2543	0.0697	0.0498	0.067*	0.402 (7)
C17A	0.2695 (15)	0.0509 (8)	−0.1310 (5)	0.074 (2)	0.402 (7)
H17A	0.1592	−0.0159	−0.1789	0.112*	0.402 (7)
H17B	0.3633	−0.0024	−0.1382	0.112*	0.402 (7)
H17C	0.2909	0.1174	−0.1665	0.112*	0.402 (7)
C16B	0.1842 (6)	0.0735 (5)	−0.0927 (4)	0.0586 (13)	0.598 (7)
H16C	0.1382	−0.0208	−0.0998	0.070*	0.598 (7)
H16D	0.1686	0.0643	−0.1806	0.070*	0.598 (7)
C17B	0.3749 (6)	0.1176 (7)	−0.0305 (6)	0.0832 (18)	0.598 (7)
H17D	0.4174	0.2156	−0.0140	0.125*	0.598 (7)
H17E	0.4386	0.0533	−0.0882	0.125*	0.598 (7)
H17F	0.3920	0.1126	0.0508	0.125*	0.598 (7)

### Atomic displacement parameters (Å^2^)

**Table:**

	*U*^11^	*U*^22^	*U*^33^	*U*^12^	*U*^13^	*U*^23^
I	0.05606 (11)	0.06525 (12)	0.03984 (9)	−0.00242 (7)	−0.00008 (6)	0.02082 (7)
Cl	0.0624 (3)	0.0565 (3)	0.0396 (3)	0.0026 (3)	0.0005 (2)	0.0076 (2)
S	0.0596 (3)	0.0483 (3)	0.0604 (3)	0.0012 (3)	0.0147 (3)	0.0339 (3)
O1	0.0480 (7)	0.0332 (7)	0.0359 (7)	0.0025 (6)	0.0051 (5)	0.0165 (5)
O2	0.0949 (15)	0.0656 (12)	0.0693 (12)	−0.0320 (11)	−0.0043 (11)	0.0322 (10)
C3	0.0463 (10)	0.0333 (10)	0.0431 (10)	0.0038 (8)	0.0085 (8)	0.0130 (8)
C1	0.0467 (10)	0.0359 (10)	0.0403 (10)	0.0077 (8)	0.0110 (8)	0.0202 (8)
C2	0.0414 (10)	0.0341 (9)	0.0392 (9)	0.0078 (8)	0.0097 (8)	0.0163 (8)
C4	0.0418 (10)	0.0425 (11)	0.0356 (9)	0.0073 (8)	0.0036 (8)	0.0090 (8)
C5	0.0496 (11)	0.0497 (12)	0.0378 (10)	0.0118 (9)	0.0089 (8)	0.0218 (9)
C6	0.0459 (10)	0.0390 (10)	0.0428 (10)	0.0083 (8)	0.0113 (8)	0.0217 (8)
C7	0.0404 (9)	0.0343 (9)	0.0358 (9)	0.0059 (7)	0.0067 (7)	0.0146 (7)
C8	0.0446 (10)	0.0359 (10)	0.0387 (9)	0.0088 (8)	0.0107 (8)	0.0199 (8)
C9	0.0414 (10)	0.0376 (10)	0.0379 (9)	0.0068 (8)	0.0071 (7)	0.0163 (8)
C10	0.0586 (13)	0.0419 (11)	0.0452 (11)	−0.0001 (10)	0.0009 (9)	0.0222 (9)
C11	0.0588 (13)	0.0525 (13)	0.0437 (11)	0.0043 (10)	0.0028 (9)	0.0273 (10)
C12	0.0428 (10)	0.0503 (12)	0.0363 (9)	0.0061 (9)	0.0046 (8)	0.0179 (9)
C13	0.0743 (16)	0.0502 (13)	0.0463 (12)	−0.0156 (12)	−0.0007 (11)	0.0216 (10)
C14	0.0800 (16)	0.0503 (13)	0.0409 (11)	−0.0101 (12)	0.0022 (11)	0.0246 (10)
C15	0.0709 (15)	0.0502 (13)	0.0551 (13)	−0.0005 (11)	0.0093 (11)	0.0306 (11)
C16A	0.076 (4)	0.041 (3)	0.050 (3)	0.005 (3)	0.002 (3)	0.025 (3)
C17A	0.112 (6)	0.057 (4)	0.062 (4)	0.028 (4)	0.015 (4)	0.033 (3)
C16B	0.084 (3)	0.044 (2)	0.051 (3)	0.017 (2)	0.019 (2)	0.0216 (19)
C17B	0.081 (4)	0.094 (4)	0.099 (4)	0.037 (3)	0.035 (3)	0.057 (3)

### Geometric parameters (Å, °)

**Table:**

I—C12	2.095 (2)	C10—C11	1.389 (3)
I—O2^i^	3.416 (2)	C10—H10	0.9300
Cl—C4	1.739 (2)	C11—C12	1.370 (3)
S—O2	1.466 (2)	C11—H11	0.9300
S—C1	1.774 (2)	C12—C13	1.378 (3)
S—C16A	1.841 (4)	C13—C14	1.381 (3)
S—C16B	1.842 (4)	C13—H13	0.9300
O1—C7	1.372 (2)	C14—H14	0.9300
O1—C8	1.379 (2)	C15—H15A	0.9600
C3—C4	1.374 (3)	C15—H15B	0.9600
C3—C2	1.397 (3)	C15—H15C	0.9600
C3—H3	0.9300	C16A—C17A	1.4807 (15)
C1—C8	1.368 (3)	C16A—H16A	0.9700
C1—C2	1.447 (3)	C16A—H16B	0.9700
C2—C7	1.386 (3)	C17A—H17A	0.9600
C4—C5	1.390 (3)	C17A—H17B	0.9600
C5—C6	1.389 (3)	C17A—H17C	0.9600
C5—H5	0.9300	C16B—C17B	1.4812 (15)
C6—C7	1.391 (3)	C16B—H16C	0.9700
C6—C15	1.495 (3)	C16B—H16D	0.9700
C8—C9	1.461 (3)	C17B—H17D	0.9600
C9—C10	1.389 (3)	C17B—H17E	0.9600
C9—C14	1.392 (3)	C17B—H17F	0.9600
			
C12—I—O2^i^	161.46 (7)	C12—C11—C10	120.4 (2)
O2—S—C1	108.18 (11)	C12—C11—H11	119.8
O2—S—C16A	126.7 (2)	C10—C11—H11	119.8
C1—S—C16A	99.2 (2)	C11—C12—C13	119.9 (2)
O2—S—C16B	96.09 (17)	C11—C12—I	121.10 (16)
C1—S—C16B	96.62 (17)	C13—C12—I	119.02 (16)
C7—O1—C8	106.81 (15)	C12—C13—C14	119.8 (2)
C4—C3—C2	116.20 (19)	C12—C13—H13	120.1
C4—C3—H3	121.9	C14—C13—H13	120.1
C2—C3—H3	121.9	C13—C14—C9	121.3 (2)
C8—C1—C2	106.99 (17)	C13—C14—H14	119.3
C8—C1—S	128.36 (16)	C9—C14—H14	119.3
C2—C1—S	124.63 (15)	C6—C15—H15A	109.5
C7—C2—C3	119.59 (18)	C6—C15—H15B	109.5
C7—C2—C1	105.15 (16)	H15A—C15—H15B	109.5
C3—C2—C1	135.26 (19)	C6—C15—H15C	109.5
C3—C4—C5	123.51 (19)	H15A—C15—H15C	109.5
C3—C4—Cl	118.70 (17)	H15B—C15—H15C	109.5
C5—C4—Cl	117.78 (17)	C17A—C16A—S	103.8 (4)
C6—C5—C4	121.43 (19)	C17A—C16A—H16A	111.0
C6—C5—H5	119.3	S—C16A—H16A	111.0
C4—C5—H5	119.3	C17A—C16A—H16B	111.0
C5—C6—C7	114.33 (19)	S—C16A—H16B	111.0
C5—C6—C15	123.3 (2)	H16A—C16A—H16B	109.0
C7—C6—C15	122.3 (2)	C17B—C16B—S	111.2 (3)
O1—C7—C2	110.77 (17)	C17B—C16B—H16C	109.4
O1—C7—C6	124.31 (18)	S—C16B—H16C	109.4
C2—C7—C6	124.93 (18)	C17B—C16B—H16D	109.4
C1—C8—O1	110.28 (16)	S—C16B—H16D	109.4
C1—C8—C9	135.71 (19)	H16C—C16B—H16D	108.0
O1—C8—C9	114.00 (16)	C16B—C17B—H17D	109.5
C10—C9—C14	117.86 (19)	C16B—C17B—H17E	109.5
C10—C9—C8	122.76 (19)	H17D—C17B—H17E	109.5
C14—C9—C8	119.37 (19)	C16B—C17B—H17F	109.5
C9—C10—C11	120.6 (2)	H17D—C17B—H17F	109.5
C9—C10—H10	119.7	H17E—C17B—H17F	109.5
C11—C10—H10	119.7		
			
O2—S—C1—C8	147.2 (2)	C2—C1—C8—O1	−0.2 (2)
C16A—S—C1—C8	−79.1 (3)	S—C1—C8—O1	−178.82 (15)
C16B—S—C1—C8	−114.1 (2)	C2—C1—C8—C9	−179.1 (2)
O2—S—C1—C2	−31.1 (2)	S—C1—C8—C9	2.3 (4)
C16A—S—C1—C2	102.6 (2)	C7—O1—C8—C1	0.6 (2)
C16B—S—C1—C2	67.5 (2)	C7—O1—C8—C9	179.74 (16)
C4—C3—C2—C7	0.8 (3)	C1—C8—C9—C10	−1.6 (4)
C4—C3—C2—C1	−179.5 (2)	O1—C8—C9—C10	179.5 (2)
C8—C1—C2—C7	−0.2 (2)	C1—C8—C9—C14	179.6 (3)
S—C1—C2—C7	178.46 (15)	O1—C8—C9—C14	0.7 (3)
C8—C1—C2—C3	−179.9 (2)	C14—C9—C10—C11	−1.3 (4)
S—C1—C2—C3	−1.2 (4)	C8—C9—C10—C11	179.9 (2)
C2—C3—C4—C5	−1.2 (3)	C9—C10—C11—C12	−1.4 (4)
C2—C3—C4—Cl	180.00 (15)	C10—C11—C12—C13	2.9 (4)
C3—C4—C5—C6	0.3 (3)	C10—C11—C12—I	−178.48 (19)
Cl—C4—C5—C6	179.08 (17)	O2^i^—I—C12—C11	−167.99 (19)
C4—C5—C6—C7	1.0 (3)	O2^i^—I—C12—C13	10.6 (4)
C4—C5—C6—C15	−178.8 (2)	C11—C12—C13—C14	−1.6 (4)
C8—O1—C7—C2	−0.7 (2)	I—C12—C13—C14	179.7 (2)
C8—O1—C7—C6	179.17 (19)	C12—C13—C14—C9	−1.1 (4)
C3—C2—C7—O1	−179.68 (18)	C10—C9—C14—C13	2.5 (4)
C1—C2—C7—O1	0.6 (2)	C8—C9—C14—C13	−178.6 (3)
C3—C2—C7—C6	0.4 (3)	O2—S—C16A—C17A	48.1 (6)
C1—C2—C7—C6	−179.3 (2)	C1—S—C16A—C17A	−72.9 (5)
C5—C6—C7—O1	178.80 (18)	C16B—S—C16A—C17A	15.4 (5)
C15—C6—C7—O1	−1.5 (3)	O2—S—C16B—C17B	−177.3 (4)
C5—C6—C7—C2	−1.3 (3)	C1—S—C16B—C17B	73.5 (4)
C15—C6—C7—C2	178.4 (2)	C16A—S—C16B—C17B	−23.2 (4)

Symmetry codes: (i) *x*+1, *y*+1, *z*+1.

### Hydrogen-bond geometry (Å, °)

**Table:**

*D*—H···*A*	*D*—H	H···*A*	*D*···*A*	*D*—H···*A*
C16A—H16B···O2^ii^	0.97	2.40	3.297 (6)	154.

Symmetry codes: (ii) −*x*, −*y*, −*z*.
